# Biopsychosocial factors of depression among community-dwelling geriatric population with low perceived social support; a population-based study

**DOI:** 10.1186/s12877-024-05211-x

**Published:** 2024-08-14

**Authors:** Nur Zahirah Balqis-Ali, Norliza Ahmad, Halimatus Sakdiah Minhat, Ahmad Zaid Fattah Azman

**Affiliations:** 1 Institute for Health Systems Research, National Institutes of Health, Ministry of Health, Selangor, Malaysia; 2 Department of Community Health, Faculty of Medicine and Health Sciences, Universiti Putra Malaysia, Serdang, Selangor Malaysia

**Keywords:** Community-dwelling geriatric population, Depression, Low social support, Prevalence, Biopsychosocial factors

## Abstract

**Background:**

Although significant and disabling consequences are presented due to geriatric population-related depression, an insufficient comprehension of various biological, psychological, and social factors affecting this issue has been observed. Notably, these factors can contribute to geriatric population-related depression with low social support. This study aimed to identify factors associated with depression among the community-dwelling geriatric population with low social support in Malaysia.

**Methods:**

This study used secondary data from a population-based health survey in Malaysia, namely the National Health Morbidity Survey (NHMS) 2018: Elderly Health. The analysis included 926 community-dwelling geriatric population aged 60 and above with low social support. The primary data collection was from August to October 2018, using face-to-face interviews. This paper reported the analysis of depression as the dependent variable, while various biological, psychological and social factors, guided by established biopsychosocial models, were the independent variables. Multiple logistic regression was applied to identify the factors. Analysis was performed using the complex sampling module in the IBM SPSS version 29.

**Results:**

The weighted prevalence of depression among the community-dwelling geriatric population aged 60 and above with low social support was 22.5% (95% CI: 17.3–28.7). This was significantly higher than depression among the general geriatric Malaysian population. The factors associated with depression were being single, as compared to those married (aOR 2.010, 95% CI: 1.063–3.803, p: 0.031), having dementia, as opposed to the absence of the disease (aOR 3.717, 95% CI: 1.544–8.888, p: 0.003), and having a visual disability, as compared to regular visions (aOR 3.462, 95% CI: 1.504–7.972, p: 0.004). The analysis also revealed that a one-unit increase in control in life and self-realisation scores were associated with a 32.6% (aOR: 0.674, 95% CI: 0.599–0.759, *p* < 0.001) and 24.7% (aOR: 0.753, 95% CI: 0.671–0.846, *p* < 0.001) decrease in the likelihood of developing depression, respectively.

**Conclusion:**

This study suggested that conducting depression screenings for the geriatric population with low social support could potentially prevent or improve the management of depression. The outcome could be achieved by considering the identified risk factors while implementing social activities, which enhanced control and self-fulfilment.

## Introduction

In recent decades, the progression of medical technology, improvements in health services, and declining fertility rates have collectively heightened the global ageing phenomenon, emphasising its significance as a priority on the international agenda [1, 2]. By 2030, approximately one-sixth of the worldwide population will be 60 or older [3]. The United Nation’s Decade of Healthy Ageing (2021–2030) initiative, spearheaded by the World Health Organization (WHO), advocates for a comprehensive, integrated, community-based approach to enhance the well-being of geriatric population, aligning with the Sustainable Development Goals’ objective of ensuring good health and well-being for all age groups [4, 5].

To effectively address the health and well-being of the geriatric population, tailored approaches that consider their diverse demographics and unique characteristics are essential. Among these subgroups, individuals with limited social support warrant particular attention. In Malaysia, research indicates that 30.8% of individuals aged 60 and above perceive their social support as inadequate [6]. Extensive evidence underscores the profound influence of social support on the geriatric population’s health outcomes [7–10]. Strong familial support correlates with enhanced well-being, reduced distress and mitigated cognitive decline [8]. Conversely, insufficient social support is linked to heightened mortality rates [10]. Furthermore, the absence of informal assistance significantly impacts health and quality of life, potentially hastening institutionalisation [9].

A significant public health concern for the geriatric population, particularly those with low social support, is the high prevalence of depression [11, 12]. In 2021, WHO reported a global significant depression prevalence of 3.8%, with the geriatric population experiencing a higher rate of 5.7% [13]. Depression has consistently ranked among the top ten leading causes of disability worldwide since 2000, making up 39% of global disability in 2019 among all mental disorders [12, 14]. According to a Malaysian population-based survey in 2018, 11.2% of the geriatric population aged 60 and above exhibited signs of depressive symptoms, with 5.3% identified as likely experiencing major depression [6]. In contrast, the lifetime prevalence of depression among the geriatric population aged 65 and above in 2011 was 2.8%, as indicated by another population-based survey [15].

A review of the aetiology and outcomes of depression reported that the incidence of suicide is nearly double among the geriatric population compared to the younger population [16]. It is linked to higher risks of illness, suicide, reduced physical and cognitive abilities, social functioning decline, and increased self-neglect—all contributing to a greater risk of mortality as compared to those without depression [16]. A cohort study of the geriatric population in the United States of America found that the geriatric population with depression had 1.2 to 1.7 higher odds of mortality in three years than the geriatric population without depression [17]. In another study, more geriatric population with depression following a myocardial infarction were found to die in the first four months after the event than those without depression [18]. Late-life depression was found to be an independent risk factor for heart failure and poor self-rated health over time [19, 20]. Regarding the economic impact, depression among the geriatric population is associated with increased medical burden, health service utilisation, more extended hospital stays, disability, and more functional impairment than most medical disorders. Specifically, Malaysia allocated and spent MYR 375 million on mental health in 2017, which accounted for 1.5% of its healthcare budget [21]. This observation highlighted the strain of mental health issues on the health system.

Examining the factors contributing to depression in the geriatric population enables the implementation of strategic management measures by addressing them. Known risk factors associated with depression among the geriatric population include being female, having physical and cognitive impairments, lacking social connections, genetic predisposition, specific personality traits, and a history of depression [22, 23]. Certain Malaysia-based studies have also regularly identified comparable risk factors among the geriatric population. These risk factors include being female, advancing age, lower educational attainment, reduced fitness, chronic illnesses, and lower functional status [15, 24–26].

Insufficient comprehensive understanding regarding the prevalence rates and factors of geriatric population-related depression with low social support has been observed when investigating this group. This research gap has been attributed to studies often exploring social support as a predictor or cause of depression rather than as the primary population of interest [7, 27, 28]. Nevertheless, depressive symptoms were significantly associated with social support. A systematic review conducted in 2019 found that various forms of social support were significantly associated with fewer depressive symptoms among the geriatric population living in Asia [7]. Reciprocating the finding, a study among the geriatric population in Hong Kong found that the depression score was lower as social support increased [29]. With regards to depression among different levels of social support, a cross-sectional study in Malaysia in 2016 found that those with lower social support had 2.7 times higher depressive scores as compared to those with high social support [24], emphasising the disparity in depression among geriatric populations with different levels of social support. With the well-established association, the current study moves away from replicating the same investigation but instead attempts to look at those with low social support as a target population.

Furthermore, a limited number of existing studies delved into an examination of the risk factors encompassed within the biopsychosocial model of depression. The model comprehensively addresses potential factors influencing depression across three primary domains: (i) biological, encompassing physiological and molecular pathology, including diseases, chronic conditions, and substance abuse; (ii) psychological, encompassing thoughts, emotions, and behaviours, including psychological distress, fear/avoidance beliefs, coping strategies, and attribution; and (iii) social, encompassing socio-economic, socio-environmental, and cultural factors such as workplace dynamics, family circumstances, and economic considerations [30, 31]. The three domains play interconnected roles in influencing a person’s vulnerability towards distress, which, if prolonged, leads towards depression [30]. Thus, identifying factors within the purview of the biopsychosocial model aids in understanding the patient’s subjective experience, impacting diagnosis, health outcomes, and compassionate care [30]. Essentially, despite effectively treating clinical or ‘biological’ symptoms, psychological and social factors significantly affect mental well-being and need to be addressed to optimise disease management [32]. Over the past three decades, extensive research has validated the model’s perspective on physical health outcomes, broadly applicable to mental health [33, 34]. Thus, this study seeks to identify critical biopsychosocial factors crucial for predicting depression among the geriatric population, emphasising targeted efforts for overall improvement. This approach prevents care fragmentation in addressing depression among the geriatric population by comprehensively considering various aspects of their lives.

Thus, the current study aims to determine the prevalence and factors associated with depression among the geriatric population with low social support in Malaysia, guided by the biopsychosocial model. This input is crucial for policymakers as it provides insights for crafting suitable interventions to enhance the well-being of this subgroup of the geriatric population. Furthermore, the prevalence and predictors of depression among the geriatric population with low social support may differ from those with better social support. Hence, they may require a different approach and strategies to be addressed. Identifying the prevalence and predictors within the subgroup population with low social support will enable the formulation of a focused and tailored action plan supported by diverse evidence [35–37]. In addition, the current study employs the biopsychosocial model to guide a comprehensive selection of various factors into consideration, offering additional wealth of knowledge to existing evidence.

## Methods

### Study design and sampling population

This cross-sectional study analysed secondary data from the National Health and Morbidity Survey 2018 (NHMS 2018): Elderly Health [6], a population-based survey among community-dwelling adults aged 50 and above in Malaysia. The survey employed a two-stage stratified cluster sampling approach, drawing on the Department of Statistics Malaysia’s sampling frame. The main stratum encompassed states and federal territories, while the secondary stratum comprised urban and rural regions within the primary stratum. This frame included 83,000 numeration blocks comprising approximately 80–120 living quarters. Respondents were randomly chosen from selected living quarters. The survey, conducted from August to October 2018, involved face-to-face interviews at the respondents’ residences. Before participation, written consent was obtained from all respondents. A comprehensive account of NHMS 2018 can be found in the published report [6].

### Target population

Data was extracted from respondents aged 60 and older to examine the well-being of the geriatric population. This selection adheres to the criteria established by the Malaysian Public Service Department, which identifies 60 as indicative of old age and retirement [38]. Within this demographic, a specific subgroup characterised by low social support was included in the analytical process. The determination of social support levels relied on respondents’ scores from the 11-item Duke Social Support Index (DSSI), assessing their perceived social support. Individuals scoring below 27 out of a maximum of 33 were classified as having low social support [39]. Consequently, the analysis included 926 complete cases involving the geriatric population aged 60 and above, identified as having low social support.

### Outcome measure

The primary focus of this study was to assess depression as the primary outcome, employing the Geriatric Depression Scale-14 (GDS-14). The NHMS 2018 opted for the GDS-14 due to its feasibility and reliability (Cronbach’s alpha 0.84), along with high sensitivity (95.5%) and specificity (84.2%) in identifying depression among the geriatric population [6, 40]. The GDS-14 employed by the NHMS 2018 was derived from the GDS-15 after a local study by Teh and Hasanah found that one of the items did not effectively distinguish between depressed and non-depressed individuals. Consequently, this item was removed from the scale [40]. Each item on the scale was scored as either ‘Yes’ or ‘No,’ indicating the presence or absence of the respective symptom. A positive response (‘Yes’) was assigned a score of one, while a negative response (‘No’) received a score of zero, resulting in a maximum possible score of fourteen. A higher total score signified a higher level of depressive symptoms. A cutoff point of six and above was selected to indicate the presence of clinically significant depression [6].

### Independent variables

The analysis selected several factors guided by the biopsychosocial model as the independent variables.

### Biological factors

The biological factors included as the independent variables in the current study were: (i) functional limitations, defined by the absence or presence of functional impairment, measured through the ability to perform activities of daily living (ADL), as measured by the 10 items Barthel index. A maximum score of 20 was categorised as the absence of functional limitation, and a total score of less than 20 indicates the presence of functional limitation [6]. The tool was tested across many studies with consistently high internal consistency (Cronbach alpha > 0.9) [41, 42]; (ii) dementia, defined by the absence or presence of the disease, as determined by the Intervention for Dementia in Elderly Africans (IDEA) Cognitive Screening tool. The tool had six items with high sensitivity (90.9%), high specificity (89.7%) in screening for dementia, and moderate internal consistency (Cronbach alpha: 0.686), as reported by a local study [43]. A total score of less than 11 out of a maximum of 15 indicates probable dementia [6]; (iii) chronic diseases, defined by the self-reported absence or presence of at least one chronic disease of either diabetes mellitus, hypertension, or hypercholesterolaemia; (iv) physical activity, categorised into either active or not active depending on respondents’ reported frequency of physical activity per week. The physical activity was measured using the Global Physical Activity Questionnaire (GPAQ), consisting of 6 items on physical activities. While the tool was yet to be piloted and validated in Malaysia, an assessment across nine countries found the tool to have moderate to substantial reliability (Kappa 0.67 to 0.73; Spearman’s rho 0.67 to 0.81) [44]. Respondents were categorised as ‘physically active’ when they met these criteria: (i) 30 min of moderate-intensity activity or walking per day on at least 5 days in a typical week; or (ii) 20 min of vigorous-intensity activity per day on at least 3 days in a typical week; or (iii) 5 days of any combination of walking and moderate- or vigorous-intensity exercise. Respondents not meeting these criteria were categorised as ‘not physically active’; v) vision disability, defined by the self-reported absence or presence of visual impairment; and vi) hearing disability, defined by the self-reported absence or presence of hearing impairment.

### Psychological factors

The psychological factors included as the independent variables in the current study were: i) control in life, defined as the perceived level of control the respondents had in intervening in their life and environment; ii) autonomy in life, defined as the perceived level of autonomy and freedom the respondents had from unwanted interference in their life; iii) satisfaction in life, defined as the perceived level of satisfaction respondents had towards their life; iv) self-realisation, defined as the perceived level of self-realisation respondents had in transcending circumstances and adopting positive outlook in their lives [45]. The control, autonomy, satisfaction in life and self-realisation were measured using the Control, Autonomy, Self-Realization and Pleasure (CASP-19) screening tool. The tool was translated in another Malaysian study, revealing strong internal consistency in both the original and translated renditions (Cronbach’s alpha > 0.8), along with satisfactory construct validity [46]. Another psychological factor considered was abuse, which was defined as the reported presence or absence of any form of abuse, including physical, psychological, financial, sexual abuse, or neglect. The National Irish Prevalence Survey on Elder Abuse was used to assess the presence of abuse [47]. The survey was piloted among 291 older people residing in an urban area of Malaysia in 2012, reporting that the adapted tool underwent content and face validity processes [48]. However, the reliability of the tool was not reported in the study. Guided by the tool, respondents were asked if they had experienced abuse in the 12 months preceding the survey. They answered ‘Yes’ or ‘No’ if they had not.

### Social factors

The social factors included as the independent variables in the current study were: i) age, categorised into several categories as follows: (a) 60–69 years old, (b) 70–79 years old, and c) > 80 years old; ii) gender, either males or females; iii) ethnicity was categorised into Malay and non-Malay groups. The non-Malay category comprised multiple ethnic groups, each with relatively small sample sizes; iv) educational level, categorised based on the highest level of education attained by respondents into no formal education, up to primary school, up to secondary school, and up to tertiary education; v) individual monthly income, defined as the monthly income received by the respondent from either work, obtained from family members, regular contributions from welfare, or irregular contributions, categorised into three levels of income based on the categorisation used by the NHMS 2018 [6]; vi) employment status, categorised into either employed or unemployed; vii) marital status, categorised into either married or single (never married, widowed, divorced); and viii) living companion, defined and classified according to whether the respondents lived alone or with others in the same household.

### Statistical analysis

The data was analysed utilising a complex sampling module, incorporating adjustments for weighting procedures that considered selection probabilities, non-response rates, strata, age, and gender. This alignment was done using the demographic distribution observed in the 2018 Malaysian population data by the Department of Statistics Malaysia [49]. The prevalence and distribution of the geriatric population with depression were described accordingly. The difference in the proportion of depression among the geriatric population with low social support was compared to the proportion of depression among the general geriatric population [6], analysed through the two-proportion Z-test. The characteristics of respondents were described according to the biological, psychological, and social factors. The weighted frequency and percentage with the 95% confidence interval (CI) were used for categorical data, while continuous data was described using the weighted mean and standard error.

Next, multiple logistic regression was performed to identify the factors associated with depression among the geriatric population with low social support. Before executing multiple logistic regression, a preliminary step involved conducting individual logistic regressions for each independent variable concerning the dependent variable. Crude odds ratios (OR) were employed to gauge the strength of these associations. The subsequent multiple logistic regression incorporated variables with a p-value < 0.25 in the initial logistic regressions [50]. The Backward LR method was then applied to select the best regression model. The outcomes were presented as Adjusted Odds Ratios (aOR) and the 95% confidence interval, with statistical significance determined at a two-tailed level of 0.05.

The correlation between variables was evaluated by examining the correlation of parameter estimates, deeming a value below 0.8 acceptable [51]. Additionally, an exploration of interaction terms was conducted. The Cox-Snell and Negelkerke R-squared values characterised the variance explained by the model. Cross-classifications illustrated the correspondence between observed and model-predicted categories for the dependent variable, offering percentage comparisons. A model with values exceeding 50% was deemed acceptable [52]. Finally, the model’s predictive performance was appraised through the Area under the Curve (AUC) and Receiver Operating Characteristic curves (ROC), where a value exceeding 0.7 denoted good accuracy [53]. All analyses were conducted using the International Business Machines Statistical Package for the Social Sciences (IBM SPSS version 29.0 (IBM Corp., Armonk, NY, USA)).

## Results

Table 1 shows the prevalence of depression among the community-dwelling geriatric population with low social support. Overall, approximately one-fifth of the geriatric population with low social support experienced depression, accounting for 22.5% (95% CI: 17.3–28.7). The overall mean score was 3.55 (SE: 0.184). A more detailed examination of the prevalence across age groups reveals an increasing trend. Specifically, the prevalence of depression among those aged 60–69 was 19.1% (95% CI: 13.7–25.9). This figure rose among older age groups, reaching 27.8% (95% CI: 18.3–39.8) for individuals aged 70–79 and escalating further to 34.1% (95% CI: 20.5–51.0) for those aged 80 and above. A comparison between urban and rural dwellers found that a slightly higher percentage of rural dwellers had depression, 25.0% (95% CI: 19.5–31.3), as opposed to those living in urban areas, 21.6% (95% CI: 15.0–29.9).

A two-proportions Z-test was performed to compare the proportions of the geriatric population with low social support who had depression (current study) with the proportion among the general older population. The prevalence of depression among the older general population was 11.2% (95% CI: 9.4–13.4), as reported in a population-based national survey in 2018 [6]. Table 2 shows that the difference was found to be significant (Z-value = -9.029, *p* < 0.001), indicating depression among the geriatric population with low social support was significantly higher than among those in the general population.

**Table 1:** Overall, by age category and by locality, the prevalence of depression among community-dwelling geriatric population with low social support

Variables	**Weighted mean score (SE)**		Depressed				Not Depressed				
		Count	Estimated population	Weighted %	95% CI		Count	Estimated population	Weighted %	95% CI	
					LL	UL				LL	UL
Overall	3.55 (0.184)	200	167,998	22.5	17.3	28.7	726	578,701	77.5	71.3	82.7
Age groups (years)											
60–69	3.28 (0.206)	111	93,763	19.1	13.7	25.9	485	397,968	80.9	74.1	86.3
70–79	4.11 (0.364)	68	55,950	27.8	18.3	39.8	198	145,450	72.2	60.2	81.7
≥ 80	3.87 (0.433)	21	18,284	34.1	20.5	51.0	43	35,282	65.9	49.0	80.0
Locality											
Urban	3.44 (0.242)	77	117,090	21.6	15.0	29.9	319	425,790	78.4	70.1	85.0
Rural	3.85 (0.162)	123	50,907	25.0	19.5	31.3	407	152,912	75.0	68.7	80.5

SE = standard error, % = percentage, CI = confidence interval, LL = lower limit, UL = upper limit

**Table 2:** Difference in proportions of having depression between geriatric population with low social support compared to the general older population

Geriatric population with low social support- Depressed					General older population – Depressed					Z-score	*p*-value
Count	Estimated population	Weighted %	95% CI		Count	Estimated population	Weighted %	95% CI			
			LL	UL				LL	UL		
200	167,998	22.5	17.3	28.7	485	346,126	11.2	9.4	13.4	-9.029	< 0.001

% = percentage, CI = confidence interval, LL = lower limit, UL = upper limit

Table 3 below describes the characteristics of the geriatric population with low social support, representing 746,699 older Malaysians. Most participants did not exhibit dementia, accounting for only 8.4% (95% CI: 6.0–11.7) affected by the condition. However, a high proportion reported having at least one chronic disease, with a prevalence of 68.7% (95% CI: 64.1–72.9). Approximately one-fifth, or 22.9% (95% CI: 18.6–27.8), of the geriatric population experienced functional limitations in ADL. The majority of respondents, aged 60 to 69, comprised 65.9% (95% CI: 60.9–70.5) of the sample. Females slightly outnumbered males at 54.9% (95% CI: 50.2–59.5). Most identified as Malay, constituting 55.5% (95% CI: 45.2–65.3), and reported being married at 62.7% (95% CI: 57.4–67.7). Over 60% had limited education, while a significant portion, 77.1%, were unemployed. Additionally, 67.3% (95% CI: 61.9–72.3) earned incomes below MYR 1000 per month. The weighted mean scores and standard errors of functional limitations, dementia, control, autonomy, self-realisation, and satisfaction with life are shown as part of Table 3.

**Table 3:** Characteristics of geriatric population with low social support based on the biopsychosocial factors (*n* = 926)

Variables	Count	Estimated population	Weighted %	95% CI		Weighted mean (SE)
				LL	UL	
**Overall**	926	746,699	100.0	-	-	
**Biological factors**						
Dementia						13.09 (0.096)^a^
No dementia	827	683,863	91.6	88.3	94.0	
Have dementia	99	62,836	8.4	6.0	11.7	
Chronic disease						
No chronic disease	319	233,697	31.3	27.1	35.9	
At least one chronic disease	607	513,002	68.7	64.1	72.9	
Functional limitation						19.35 (0.075)^b^
No limitation	742	575,783	77.1	72.2	81.4	
Impaired	184	170,916	22.9	18.6	27.8	
Physical activity						
Active	553	472,865	63.3	58.0	68.3	
Not Active	373	273,834	36.7	31.7	42.0	
Hearing disability						
No disability	864	687,701	92.1	87.9	94.9	
Impaired	62	58,998	7.9	5.1	12.1	
Visual disability						
No disability	864	698,834	93.6	90.7	95.6	
Impaired	62	47,864	6.4	4.4	9.3	
**Psychological factors**						
Abuse						
Absence	782	624,856	83.7	77.5	88.4	
Presence	144	121,843	16.3	11.6	22.5	
Perceived control in life	926	746,699				8.52 (0.238)^c^
Perceived autonomy in life	926	746,699				11.61 (0.216) ^c^
Satisfaction with life	926	746,699				11.61 (0.236) ^c^
Self-realisation	926	746,699				11.24 (0.176) ^c^
**Social factors**						
Age group (years)						
60–69	596	491,732	65.9	60.9	70.5	
70–79	266	201,401	27.0	22.9	31.4	
≥ 80	64	53,566	7.2	5.6	9.2	
Gender						
Male	429	336,938	45.1	40.5	49.8	
Female	497	409,761	54.9	50.2	59.5	
Ethnicity						
Malay	589	414,208	55.5	45.2	65.3	
Non-Malay	337	332,492	44.5	34.7	54.8	
Highest education level						
No formal education	204	127,856	17.1	13.6	21.3	
Primary education	478	345,202	46.2	39.8	52.8	
Secondary education	201	226,511	30.3	25.2	36.1	
Tertiary education	43	47,129	6.3	4.1	9.7	
Monthly individual income						
< MYR 1000	669	502,567	67.3	61.9	72.3	
MYR 1000–1999	172	148,836	19.9	16.2	24.3	
≥ MYR 2000	85	95,296	12.8	9.0	17.9	
Employment status						
Employed	247	171,221	22.9	19.8	26.4	
Unemployed	679	575,478	77.1	73.6	80.2	
Marital status						
Married	589	468,154	62.7	57.4	67.7	
Single (never married/widowed/divorcee)	337	278,545	37.3	32.3	42.6	
Living companion						
Lives with other people	847	689,402	92.3	90.0	94.1	
Lives alone	79	57,297	7.7	5.9	10.0	

% = percentage, SE: Standard error, CI = confidence interval, LL = lower limit, UL = upper limit, MYR = Malaysian Ringgit

a = measured by Intervention for Dementia in Elderly Africans (IDEA) tool, b = measured by Barthel’s index, c = measured by Control, Autonomy, Self-Realization, and Pleasure (CASP-19) tool

Multiple logistic regression was performed to identify the biopsychosocial factors of depression among the geriatric population with low social support, as shown in Table 4. The model found two biological factors associated with depression among the geriatric population with low social support. The geriatric population who had dementia had a 3.7 times higher likelihood of developing depression as compared to those without the disease (aOR 3.717, 95% CI: 1.554–8.888, p: 0.003). The geriatric population with visual disability had a 3.5 times higher likelihood of developing depression than those without the disability (aOR 3.462, 95% CI: 1.504–7.972, p: 0.004).

Two continuous psychological variables, control in life and self-realisation, were statistically significant in the multivariable analysis. For every one-unit increase in control in life score, the odds of developing depression were decreased by 32.6% (aOR: 0.674, 95% CI: 0.599– 0.759, *p* < 0.001). Likewise, an increase in one unit in self-realisation score decreased the likelihood of developing depression by 24.7% (aOR: 0.753, 95% CI: 0.671–0.846, *p* < 0.001). The only social factor found to be significantly associated with depression was marital status, with those who were single having a 2.0 times higher likelihood of developing depression than those who were married (aOR 2.010, 95% CI: 1.063–3.803, p: 0.031).

The model accounted for the variance in depression, ranging between 34.0% (Cox and Snell R Square) and 51.8% (Nagelkerke R Square). It demonstrated an 84.6% accuracy in correctly classifying depressive status. The ROC curve for the final model displayed a statistically significant difference (*p* < 0.001), with an area under the curve of 85.8% (0.858, 95% CI: 0.830–0.885).

**Table 4:** Logistic regression model for factors associated with depression among geriatric population with low social support (*n* = 926)

Variables	OR	95% CI for Exp(B)		*p*-value	aOR	95% CI for Exp(B)		*p*-value
		Lower	Upper			Lower	Upper	
**Biological factors**								
Dementia								
No dementia (ref)								
Have dementia	5.040	2.685	9.460	< 0.001	3.717	1.554	8.888	0.003**
Visual disability								
No disability (ref)								
Impaired	6.095	3.130	11.871	< 0.001	3.462	1.504	7.972	0.004**
Hearing disability								
No disability (ref)								
Impaired	6.087	2.548	14.541	< 0.001				
Chronic disease								
No chronic disease (ref)								
At least one chronic disease	1.447	0.908	2.307	0.119	1.441	0.827	2.511	0.191
Functional limitation								
No limitation (ref)								
Impaired	3.796	2.152	6.695	< 0.001	1.565	0.764	3.206	0.222
Physical activity								
Active (ref)								
Not Active	3.280	1.885	5.705	< 0.001	1.410	0.809	2.458	0.262
**Psychological factors**								
Abuse								
Absence (ref)								
Presence	1.352	0.670	2.729	0.396				
Perceived control in life	0.595	0.538	0.658	< 0.001	0.674	0.599	0.759	< 0.001***
Self-realisation	0.680	0.606	0.762	< 0.001	0.753	0.671	0.846	< 0.001***
Perceived autonomy in life	0.746	0.655	0.850	< 0.001	0.976	0.847	1.126	0.741
Perceived satisfaction with life	0.613	0.538	0.700	< 0.001				
**Social factors**								
Marital status								
Married (ref)								
Single (never married/widowed/divorcee)	2.358	1.429	3.890	0.001	2.010	1.063	3.803	0.031*
Highest education level								
No formal education (ref)								
Primary education	0.861	0.532	1.393	0.042	1.404	0.758	2.600	0.145
Secondary education	0.395	0.171	0.915	0.085	1.415	0.598	3.345	0.297
Tertiary education	0.339	0.119	0.962	0.804	2.582	0.701	9.512	0.350
Monthly individual income								
< MYR 1000 (ref)								
MYR 1000–1999	0.657	0.276	1.565	0.003	1.136	0.537	2.403	0.279
≥ MYR 2000	0.258	0.107	0.622	0.128	0.603	0.256	1.416	0.294
Age								
60–69 (ref)								
70–79	1.633	0.882	3.022	0.045	0.692	0.393	1.220	0.711
≥ 80	2.200	1.019	4.748	0.450	0.812	0.281	2.343	0.763
Gender								
Male (ref)								
Female	1.057	0.653	1.710	0.821				
Employment status								
Employed (ref)								
Unemployed	1.632	1.025	2.598	0.039				
Living companion								
Lives with other people (ref)								
Lives alone	0.816	0.403	1.655	0.570				
Ethnicity								
Malay (ref)								
Non-Malay	0.699	0.410	1.193	0.187				

OR = odd ratio, aOR = adjusted odd ratio, CI = confidence interval

Backward LR was applied

Correlation and interaction terms were checked

Classification table (overall percentage: 84.6%), Cox and Snell R squared (0.340), Nagelkerke R squared (0.518), ROC = 0.858.

*p < 0.05, ** *p* < 0.01, ***p < 0.001

## Discussion

The prevalence of depression among the geriatric population residing in the community with low social support in Malaysia was found to be 22.5%, encompassing approximately one-fifth of this demographic. Notably, this rate was significantly higher than the prevalence of depression observed in the broader community-dwelling geriatric population aged above 60 in Malaysia, which was documented at 11.2% [6]. This discrepancy underscores the heightened concern for depression within the specific subgroup characterised by low social support, in contrast to the general geriatric population. The prevalence was also higher compared to another study among the community-dwelling geriatric population in Malaysia by Vanoh et al. [25], which found the prevalence to be 16.5%. Nonetheless, it is essential to note that this study utilised GDS-14 to assess depression, differing from the GDS-15 employed in the referenced study, potentially impacting the observed distinctions. A study conducted in Penang among the geriatric population aged above 60 receiving financial aid reported a notably high depression prevalence of 56.1% [24]. Furthermore, a study involving a geriatric population over 60 residing in rural areas documented an even higher prevalence, reaching 85.5% [26], echoing the current study’s finding whereby the prevalence of depression is higher among rural dwellers. These variations underscore the significance of recognising diverse prevalence rates of depression among distinct subgroups of the geriatric population, emphasising the need for tailored attention to manage the disease effectively.

In contrast to the global prevalence, this study identified a higher prevalence of depression among individuals aged above 60 compared to the 2021 WHO report, which documented a prevalence of 5.7% [13]. However, the prevalence is lower when compared with the results of a 2021 meta-analysis reporting a 31.4% prevalence of depression among the geriatric population from 23 countries worldwide [54]. Discrepancies in prevalence rates among this study, the WHO report, and the meta-analysis may be attributed to variations in sample size, study subjects, depression severity, study location, and the measurement tools utilised across the studies.

Upon further analysis based on age groups, the prevalence of depression exhibited an upward trend with increasing age. Notably, Abdul Rashid-Tahir’s study similarly found a higher prevalence of severe depressive symptoms in older age groups, where the prevalence rates were reported as 16.0%, 22.1%, and 34.0% among the age groups 60–69, 70–79, and ≥ 80, respectively. [24]. This suggests a heightened concern that demands a more vigorous management approach, particularly for the older demographic.

The presence of dementia emerged as a contributing factor to depression in community-dwelling geriatric populations with low social support. This observation aligns with a German study involving 3327 geriatric population attending outpatient services, revealing that those aged above 75 with multi-domain cognitive impairment with amnesia (aOR = 2.5, 95% CI = 1.62, 4.00, *p* < 0.001) and those with multi-domain cognitive impairment without amnesia (aOR = 2.3, 95% CI = 1.37, 3.95, *p* = 0.002) had a significantly higher likelihood of experiencing depression than those without cognitive impairment [55]. Similarly, a study involving 2005 geriatric population receiving financial aid in Penang, Malaysia, found that geriatric population with cognitive impairment had a 2.5 times higher likelihood of developing depression compared to those without (aOR = 2.5, 95% CI = 1.3, 4.6, *p* < 0.001) [24]. However, in a 2013 study involving a 2264 community-dwelling geriatric population in Malaysia, cognitive impairment was found to be significantly associated with depressive disorder but was not identified as a determinant of the disease [25]. It is important to note that the tools used to measure cognitive function and depression varied across the different studies conducted in Malaysia. While depression has been identified as a factor contributing to cognitive decline primarily through inflammatory responses in the brain [56], the presence of dementia or cognitive impairment itself can lead to or worsen depression. As per the cognitive neuropsychological model of depression, negative affect in individuals with dementia plays a central role in the development of depression [57]. Additionally, several studies have implicated a genetic predisposition in linking cognitive impairment with depression [58]. Despite persistent efforts to explain the causal relationship, preventing cognitive decline through effective methods such as hormone therapies, physical exercise, cognitive training, and a healthy diet may play a crucial role in managing depression among the geriatric population [59].

Experiencing a visual disability emerged as another biological factor influencing depression among the geriatric population with low social support in this study. Similarly, research involving 624 community-dwelling individuals aged 57 and above in the Netherlands revealed a significant correlation between visual disability and elevated depressive symptoms (*r* = 0.162; *p* < 0.05) [60]. Furthermore, a study encompassing 13,900 geriatric population in the United Kingdom demonstrated that those with visual impairment had a 2.7 times higher likelihood of experiencing depression compared to those without impairment (aOR: 2.69, 95% CI, 2.03, 3.56) [61]. Visual disability poses various challenges for the geriatric population regarding functioning and social engagement, limiting their participation with family and friends and reducing social interactions [62]. Consequently, the geriatric population may experience feelings of loneliness, hopelessness, and exclusion, heightening the risk of developing depression [62].

The degree to which the geriatric population perceived control in their lives emerged as a significant factor associated with depression in this study. The higher an older person perceives their ability to control their life outcomes, the lower the risk of developing depression. This finding resonates with a New Zealand study involving 1489 individuals aged above 65, where increasing scores in perceived control in life were associated with a reduced likelihood of developing depression (β= -0.34, *p* < 0.05) [63]. While the precise mechanism linking perceived control in life to depression is not yet conclusively established, some theories suggest that the ability to control life outcomes enhances the overall psychological well-being of the geriatric population [64]. Additionally, studies have indicated that perceived control moderates the impact of financial strain on psychological distress, as well as the relationship between late-life stressors and depressive symptoms [63, 65].

Self-realisation, characterised as an older person’s psychological ability to transcend circumstances and adopt a positive outlook, acknowledging personal merits, viewing ageing as a natural process, and perceiving life as fulfilling, meaningful, complete, and joyful [66], was another factor found to be associated with depression among the geriatric population with low social support in this study. A 2014 meta-analysis indicated that self-realisation is among the characteristics promoting self-care, thereby improving health and well-being among the geriatric population [67].

Marital status emerged as the sole social factor significantly linked to depression. This finding aligns with a Malaysian study involving the rural-dwelling geriatric population, which reported that being single or widowed increased the likelihood of depression compared to being married (OR: 3.27, 95% CI: 1.66, 6.44, *p* < 0.05) [68]. However, a separate Malaysian study among the geriatric population receiving financial aid in Penang found that being married elevated the likelihood of developing depression compared to being single (aOR: 10.5, 95% CI: 5.40, 20.5, *p* < 0.001) [24]. Another Malaysian study of a community-dwelling geriatric population found no association between marital status and depression [25]. Despite variations in findings, the presence of a spouse has been theorised to play a protective role against depression through the psychological support provided, especially during stressful events [69]. The absence or loss of a spouse often translates to reduced support and diminished control over life events, resulting in decreased motivation to navigate through the ensuing days [70]. Furthermore, individuals who experienced spousal loss at an older age were reported to have lower life satisfaction, primarily due to loneliness, further contributing to depression [70].

It is noteworthy that several factors examined and significantly linked to depression among the geriatric population in previous studies conducted in Malaysia, such as gender, ethnicity, income level, and functional impairments [15, 24–26], did not demonstrate significance in this study. Differences may be affected by the variations observed in measurement tools and cut-off values utilised across studies. However, the lack of significance of these factors in this study may also be attributed to other factors. For instance, concerning gender, while existing research suggests that females are more susceptible to mental illness due to cultural and societal expectations, males are less inclined to seek treatment for mental health issues, with a higher suicide rate identified among them [71]. These factors may have balanced the prevalence of depression among each gender, rendering the absence of a significant association between gender and depression. Otherwise, these differences may indicate that the geriatric population with low social support experiences a unique influence on depression due to distinct differences in their perceptions, behaviours and environment compared to the general geriatric population.

### Implications for practice and future research

Considering the high prevalence of depression among the geriatric population with low social support, it would be beneficial for relevant stakeholders and policymakers to consider implementing targeted programs addressing this issue. For instance, given that dementia and vision disability were identified as factors, implementing care initiatives that promote cognitive activity, such as engaging in cognitively stimulating games and assisting individuals with visual impairments in household tasks, could positively impact their emotional well-being [72–74]. Additionally, recognising the significance of control and autonomy in life as factors contributing to depression among the geriatric population with low social support, the introduction of community-based social support programs empowering the geriatric population to actively participate, contribute, and take leadership roles in various community projects could play a crucial role in preventing and managing symptoms associated with depression in this demographic. These programs encompass support groups, recreational activities, and community events, fostering a sense of belonging and control in life [75].

Identifying factors across the three biological, psychological, and social domains is paramount. These findings advocate for a shift in the paradigm of depression management, especially for the geriatric population with limited social support. The approach should transcend the conventional focus solely on treating clinical symptoms and instead adopt a holistic perspective that addresses various facets of an older person’s life [30, 31]. When treating such patients, it becomes imperative to look beyond clinical symptoms. This involves evaluating the presence of support in their home environment, assessing the requirement for assistance in daily functions, and exploring additional forms of care. Any identified needs should be thoroughly evaluated and referred to relevant health or social care providers appropriately.

For healthcare providers, the findings underscore the significance of adopting a more vigilant and comprehensive approach to depression screening, particularly among the geriatric population with low social support. When conducting screenings for depression or addressing other health concerns in the geriatric population, the insights derived from the findings highlight the necessity for heightened awareness regarding additional risk factors. Specifically, healthcare providers should pay attention to the potential presence of depression in the geriatric population with cognitive impairment, visual disabilities, those without a spouse or partner, and those expressing a sense of incapacity in managing various aspects of their lives. This necessitates a proactive and thorough exploration of the potential depression in these geriatric populations. Lastly, initiatives such as health education, promotion, and awareness programs targeting caregivers of geriatric population with low social support regarding the risk of depression should be implemented to ensure caregivers are equipped to address the needs of the geriatric population effectively [75]. Finally, this study was grounded in Malaysia’s distinct cultural traditions and healthcare infrastructure, offering insights that may resonate with nations sharing similar contextual backgrounds.

### Strengths and limitations

The study employed a community-based population survey with a substantial sample of the community-dwelling geriatric population. Although previous studies in Malaysia have explored the prevalence and depression among the geriatric population, this study uniquely focused on a subgroup with low social support. The findings provide valuable insights into the distinct prevalence and severity of depression within this subgroup, shedding light on the factors of depression among them. Notably, the study stands out as one of the few guided by the biopsychosocial framework of depression, delivering comprehensive insights into all critical dimensions influencing depression development. It is important to acknowledge certain limitations, such as the exclusion of institutionally residing geriatric populations potentially with more severe depression. In the current study, certain variables such as age and gender were classified as social rather than biological factors. This approach was based on consideration of the impact of socio-cultural views towards differing age and gender rather than the biological changes. Nevertheless, different studies may classify the same factors under differing domains. Additionally, the study’s cross-sectional nature limits the ability to establish causation between relevant variables. Furthermore, the depression tool and other survey instruments in NHMS 2018 relied on respondents’ perceptions, introducing the potential for individual bias.

## Conclusion

The primary objective of this study was to determine the prevalence and factors associated with depression among the geriatric population with low social support in Malaysia, guided by the biopsychosocial model of depression. In summary, the study revealed a substantial prevalence of depression among the geriatric population with low social support, reaching 22.5%, equivalent to approximately one-fifth of this demographic. This prevalence is notably higher than that observed in the older population, raising significant concerns. The factors of depression among the geriatric population with low social support spanned biological, psychological and social aspects. To address this issue effectively, depression screening for the geriatric population should encompass a comprehensive assessment of these factors. Management strategies should prioritise the thorough evaluation of social support and assistance to the geriatric population with identified risk factors, enabling them to navigate and perform various functions within their home and community. The introduction of community-based social activities to improve the perception of control and self-realisation may prevent or improve the management of depression. Collaborative referrals to relevant providers for tailored care should be vital to these management strategies.

## Data Availability

The data that support the findings of this study are available from the Sector for Biostatistics & Data Repository, National Institute of Health, Ministry of Health Malaysia. However, restrictions apply to the availability of these data, which were used under license for the current study and are not publicly available. Data are, however, available upon reasonable request from Dr Mohd Azahadi Omar (drazahadi@moh.gov. my), the head of the sector for Biostatistics & Data Repository, National Institute of Health, Ministry of Health Malaysia and with permission of the Director-General of Health, Malaysia.

## Abbreviations

WHO: The World Health Organization
NHMS: The National Health Morbidity Survey, Malaysia
OR: Odd Ratio
SE: Standard error
CI: Confidence Interval
MYR: Malaysian Ringgit
ADL: Activities of daily living
DSSI: The Duke Social Support Index
QoL: Quality of life
GDS: Geriatric Depression Scale
CASP-19: Quality of Life scale of Control, Autonomy, Self-realization, and Pleasure
IDEA: Intervention for Dementia in Elderly Africans Cognitive Screen
AUC: Area under the curve
ROC: Receiver operating characteristic
MREC: Medical and Research Ethics Committee
NMRR: National Medical Research Register
